# Supplementary material for: Rabies seropositive individuals, dogs, and healthcare professionals without prior vaccination in four Brazilian Indigenous communities
Source: PLoS Negl Trop Dis. 2025 Jan 31;19(1):e0012850. doi: 10.1371/journal.pntd.0012850 (PMC11798433; doi:10.1371/journal.pntd.0012850)
Supplement: S1 Fig — (PDF) [file pntd.0012850.s001.pdf]

S1 Fig. Epidemiological questionnaire for identification and general knowledge of all study participants.

Indigenous Epidemiological File I

Name of interviewer: ________________Date: ___/__/___Place of collection: ________________

1.Name:	2. Place of birth:
3. Date of birth:	4. Gender: ( ) female ( ) male ( ) Other______________
5. Ethnicity:	6. Subgroup:

7. Education

( ) No literacy ( ) Incomplete elementary school ( ) Complete secondary school ( ) Incomplete elementary school ( ) Complete elementary school ( ) Incomplete higher education

( ) Complete primary school ( ) Incomplete secondary school ( ) Complete higher education

8. Occupation:	9. Number of people in the house:

10. Fixed in the indigenous village or moves around:

11. Do you drink alcohol? ( ) Yes ( ) No	12. Uses tobacco (cigarette/pipe) ( ) Yes ( ) No

13. Source of drinking water: ( ) River ( ) Spring ( ) PipedOther:

14. Disposal of faeces: ( ) Septic tank ( ) Dry pit ( ) EnvironmentOther:_____

15. Do you hunt: ( ) Yes ( ) No	16. Which animal do you usually hunt: ( ) Armadillo ( ) Skunk ( ) Paca ( ) Wild boar Other:______
17. Do you eat game ( ) Yes ( ) No	18. Point of meat: ( ) raw ( ) rare ( ) well-done
19. If female, are you pregnant? () Yes ( ) No.	20. If female, how many children?

21. If you are a woman, have you ever had a miscarriage? ( )Yes Several times?_____( ) No

22. Do you have pets? ( ) Yes ( ) dogs, how many?_______________( ) Cats, how many? _____________________( ) Other. Which?_____________________( ) No

23. Are the animals vaccinated for rabies? ( ) Yes, when was the last time?_________Do you vaccinate periodically?_____________( ) No

24. Are the animals vaccinated against other diseases? ( ) Yes, for which ones? _______________ When was the last time you vaccinated?___________Do you vaccinate periodically?_________( ) No

25. Where the animals live: ( ) home ( ) peridomicile ( ) community

26. Rodent sightings: ( ) Yes ( ) No.	25. Rodent viewing period: ( ) Day Night ( )
27. Do you have vision difficulties: ( ) Yes ( ) No	28. Have you ever been bitten by: ( ) flea ( ) tick Other:______
29. Have you ever had foot problems: ( ) Yes ( ) No	30. Have you ever seen a bat near the village? ( ) Yes ( ) No
31. Have you ever come into contact with a bat: ( ) Yes ( ) No	32. Have you ever seen pets in contact with bats? ( ) Yes ( ) No

33. Comments:
