# Supplementary material for: Rabies seropositive individuals, dogs, and healthcare professionals without prior vaccination in four Brazilian Indigenous communities
Source: PLoS Negl Trop Dis. 2025 Jan 31;19(1):e0012850. doi: 10.1371/journal.pntd.0012850 (PMC11798433; doi:10.1371/journal.pntd.0012850)
Supplement: S2 Fig — (PDF) [file pntd.0012850.s002.pdf]

S2 Fig. Epidemiological questionnaire to identify risk factors for participants with soroneutralizing antibodies against rabies.

Indigenous Epidemiological File II

Name of interviewer: ________________Date: ___/___/___Place of collection: _____________

1. Name:	2. Date of birth:
3. Ethnicity:	4. Gender: ( ) Female ( ) Male ( ) Other__________________
5. Occupation:	6. Do you have a vaccination card? ( ) Yes ( ) No
7. Do you know what zoonosis is? ( ) Yes ( ) No	8. Have you ever heard of rabies? ( ) Yes ( ) No
9. Do you know which animals transmit rabies? ( ) No ( ) Yes, which ones?<br>( ) Dog ( ) Bat ( ) Cat ( ) Other: ________________	10. Do you keep animals? If so, which one?<br>______________
11. Have you ever seen bats in the village? ( ) Yes ( ) No	12. Where have you seen bats? __________
13. Do you still visit the place where you last saw bats? ( ) Yes ( ) No	
14. Have you ever seen a dead bat in the village? ( ) Yes, how many times? ______________( ) No	
15. What would you do if you found a dead bat in the village? __________________________	
16. Have you seen any other wild animals in the village? ( ) Yes, ( ) monkey ( ) fox ( ) wolf ( ) bush dog ( ) capybara ( ) other:____________________( ) No	
17. Have you ever seen pets in contact with wild animals? ( ) Yes ( ) No	
18. Have you ever observed bites or scratches on pets? ( ) Yes ( ) No	
19. What do you do when you see bites or scratches on pets in the village?<br>____	
20. Can wild animal bites or scratches on domestic animals mean a risk of disease infection for them? ( ) Yes ( ) No	
21. Have you ever seen a pet hunting wild animals? ( ) Yes ( ) No	
22. Do the dogs in the village eat the carcasses of other animals? ( ) Yes ( ) No	
23. Do the animals in the village have a veterinary service? ( ) Yes ( ) No	
24. If an animal needs professional care, what is done? ________________________	
25. Have you ever seen animal bites or scratches on children in the village? ( ) Yes ( ) No	
26. Hunting habit: ( ) Yes ( ) No	27. Have you ever eaten wild animal meat? ( ) Yes ( ) No
28. Have you ever been ill and not sought medical help? ( ) Yes ( ) No	29. Have you ever experienced the following symptoms simultaneously? ( ) general malaise ( ) fever ( ) headache ( ) nausea ( ) sore throat ( ) muscle pain ( ) irritability ( ) restlessness
30. Do you self-medicate? ( ) Yes ( ) No	31. In the event of a medical emergency, how quickly can you get professional help? ____________________
32) Have you ever heard of human rabies in the village?<br>( ) Yes, how often? _____________( ) No	33. Have you ever heard of a suspected rabies death in the village? ( ) Yes ( ) No
34. Remarks by the interviewer or interviewee:<br>____	
