# Supplementary material for: Rabies seropositive individuals, dogs, and healthcare professionals without prior vaccination in four Brazilian Indigenous communities
Source: PLoS Negl Trop Dis. 2025 Jan 31;19(1):e0012850. doi: 10.1371/journal.pntd.0012850 (PMC11798433; doi:10.1371/journal.pntd.0012850)
Supplement: Attachment — Submitted filename: Response letter_Rabies 19.12.docx [file pntd.0012850.s004.docx]

Botucatu, December 19^th^, 2024

**Response letter**

Section Editor

Re: Decision on your PLOS Neglected Tropical Diseases submission (PNTD-D-24-00608R2) - [EMID:f40914fdf8beb56d]

Dear Nithya,

I am sharing with you our reviewed manuscript entitled “"One Health approach to Rabies: seropositive individuals, dogs, and healthcare professionals without prior vaccination in Brazilian Indigenous communities”. All the corrections and suggestions indicated by the reviewers were fully addressed, accepted, and rewritten.

A response letter has been added to identify the editor's and reviewers' requests, with a detailed list of the corrections and a description of the changes made to the manuscript. To better identify the corrections, our comments are in italics and the changes in red (as in the manuscript corrected). Thank you for the opportunity and please do not hesitate to contact us for any further clarification.

Sincerely,

Jane Megid, DMV, MSc, PhD.

E-mail: jane.megid@unesp.br

Manuscript ID: PNTD-D-24-00608R2


Dear Dr Megid,

Thank you for submitting your paper, "Rabies seropositive individuals, dogs, and healthcare professionals without prior vaccination in four Brazilian Indigenous communities", to PLOS Neglected Tropical Diseases. We have checked over your submission and, before we can assign your manuscript to the Editor, we ask that you first please make the following modifications:

1) We note that you have uploaded "Copyright PLOS_Rabies 26.09.docx" as Supporting Information File. Please change the file type to Other.

**Authors' comments:** *Thank you for the observation. Now the file was at Other’s type.*

2) Some material included in your submission may be copyrighted. According to PLOS’s copyright policy, authors who use figures or other material (e.g., graphics, clipart, maps) from another author or copyright holder must demonstrate or obtain permission to publish this material under the Creative Commons Attribution 4.0 International (CC BY 4.0) License used by PLOS journals. Please closely review the details of PLOS’s copyright requirements here: PLOS Licenses and Copyright. If you need to request permissions from a copyright holder, you may use PLOS's Copyright Content Permission form.

Please respond directly to this email and provide any known details concerning your material's license terms and permissions required for reuse, even if you have not yet obtained copyright permissions or are unsure of your material's copyright compatibility. Once you have responded and addressed all other outstanding technical requirements, you may resubmit your manuscript within Editorial Manager.

Potential Copyright Issues:

[Figure1]: In the manuscript Figure1 legend, you have mentioned the source details only as " Collection 8 of the Annual Land Use Land Cover Maps of Brazil, accessed on 2023-08-11  <https://brasil.mapbiomas.org/en/colecoes-mapbiomas/> Country Boundaries - World Bank Official Boundaries. Data source and copyright link: <https://datacatalog.worldbank.org/search/dataset/0038272/World-Bank-Official-Boundaries>"

but In the  "Copyright PLOS_Rabies 26.09.docx" file you have mentioned the source details as below:

1)  copyright link: <https://brasil.mapbiomas.org/en/faq/como-faco-para-citar-os-dados-do-mapbiomas/>
data source link: <https://brasil.mapbiomas.org/en/colecoes-mapbiomas/>

Country Boundaries - World Bank Official Boundaries
Data source and copyright link: <https://datacatalog.worldbank.org/search/dataset/0038272/World-Bank-Official-Boundaries>

2) State Limit and Municipality - Public Continuous cartographic bases IBGE - Brazil

copyright and technical information link:
<https://www.ibge.gov.br/en/geosciences/maps/continuous-cartographic-bases/18067-continuous-cartographic-bases-brazil.html?lang=en-GB&t=sobre>
<https://www.ibge.gov.br/en/access-to-information/institutional/the-ibge.html>
data source link: <https://www.ibge.gov.br/en/geosciences/maps/continuous-cartographic-bases/18067-continuous-cartographic-bases-brazil.html?lang=en-GB>

3) Indigenous Land and Community locations
data copyright link (unrestricted public data): <https://www.gov.br/funai/pt-br/acesso-a-informacao/informacoes-classificadas>
data source link: <https://geoserver.funai.gov.br/geoserver/web/wicket/bookmarkable/org.geoserver.web.demo.MapPreviewPage?1&filter=false>

Please be so kind and specifically confirm the source details. [please confirm which of these two  source details is appropriate for this submission?

**Authors' comments:** *We appreciate the doubt and apologize for the error. Now, we specify all data in the manuscript.*

**Now you read: “**CC BY 4.0 license - **Map created by the authors, using the following data:**

1. **State and Municipality Boundaries**

- **Data Source**: Public Continuous Cartographic Bases from IBGE – Brazil
- **Data Source Link**: IBGE Continuous Cartographic Bases - <https://www.ibge.gov.br/en/geosciences/maps/continuous-cartographic-bases/18067-continuous-cartographic-bases-brazil.html?lang=en-GB&amp;t=sobre> ; Continuous Cartographic Bases - IBGE  - <https://www.ibge.gov.br/en/geosciences/maps/continuous-cartographic-bases/18067-continuous-cartographic-bases-brazil.html?lang=en-GB&amp;t=sobre>
- **License**: Public Open Data
- **Access Date**: 2024/01/17
- **Copyright and Technical Information**: IBGE Institutional Information - <https://www.ibge.gov.br/en/access-to-information/institutional/the-ibge.html>

2. **Indigenous Land and Community Locations**

- **Data Source**: FUNAI Geoserver
- **Data Source Link**: FUNAI Geoserver Map Preview - <https://geoserver.funai.gov.br/geoserver/web/wicket/bookmarkable/org.geoserver.web.demo.MapPreviewPage?1&amp;filter=false>
- **License**: Public Open Data
- **Access Date**: 2024/01/17
- **Copyright Information**: Unrestricted Public Data - FUNAI - <https://www.gov.br/funai/pt-br/acesso-a-informacao/informacoes-classificadas>

3. **Mapbiomas 2020 Land Cover (Water and Vegetation)**

- **Data Source**: MapBiomas Project - Collection 7 of the Annual Land Use Land Cover Maps of Brazil
- **Data Source Link**: <https://storage.googleapis.com/mapbiomas-public/initiatives/brasil/collection_7/lclu/coverage/brasil_coverage_2020.tif>
- **License**: CC BY
- **Access Date**: 2024/01/17
- **Copyright and Technical Information**: MapBiomas FAQ - How to Cite - <https://brasil.mapbiomas.org/en/faq/como-faco-para-citar-os-dados-do-mapbiomas/>” *Please see lines 176-209.*

2)  [Copyright PLOS_Rabies 26.09.docx] contains branding/a logo. We are not permitted to publish this under our CC BY 4.0 license, even with permission. We ask that you please remove or replace it.

**Authors' comments:** *We removed the logo. Thank you for the correction.*

Once you have made the necessary changes in Editorial Manager, please re-approve the submission by following the same steps as before.

Please resolve these issues and resubmit your manuscript within 30 days. If you need more time, please email the journal office at [plosntds@plos.org](mailto:plosntds@plos.org). We are happy to grant extensions of up to one month past this due date. If we do not hear from you within 30 days, we will withdraw your manuscript on your behalf.                                                                               

If you have any questions or require assistance, please don't hesitate to get in touch. To help us minimize inbox volumes, we kindly ask that you not reply to this email simply to confirm you have resubmitted your revisions.

Kind regards,

Nithya Venkatesan
PLOS Neglected Tropical Diseases
[plosntds@plos.org](mailto:plosntds@plos.org)

Author guidelines: <https://journals.plos.org/plosntds/s/submission-guidelines>

__________________________________________________
