# Supplementary material for: Rabies seropositive individuals, dogs, and healthcare professionals without prior vaccination in four Brazilian Indigenous communities
Source: PLoS Negl Trop Dis. 2025 Jan 31;19(1):e0012850. doi: 10.1371/journal.pntd.0012850 (PMC11798433; doi:10.1371/journal.pntd.0012850)
Supplement: Attachment — Submitted filename: Response letter_Rabies 16.09.docx [file pntd.0012850.s003.docx]

Botucatu, September 16^th^, 2024

**Response letter**

To: Victoria J. Brooks
Section Editor

Re: Decision on your PLOS Neglected Tropical Diseases submission (PNTD-D-24-00608) - [EMID:f40914fdf8beb56d]

Dear Editor,

I am sharing with you our reviewed manuscript entitled “"One Health approach to Rabies: seropositive individuals, dogs, and healthcare professionals without prior vaccination in Brazilian Indigenous communities”. All the corrections and suggestions indicated by the reviewers were fully addressed, accepted, and rewritten.

A response letter has been added to identify the editor's and reviewers' requests, with a detailed list of the corrections and a description of the changes made to the manuscript. To better identify the corrections, our comments are in italics and the changes in red (as in the manuscript corrected). Thank you for the opportunity and please do not hesitate to contact us for any further clarification.

Sincerely,

Jane Megid, DMV, MSc, PhD.

E-mail: jane.megid@unesp.br

Reviewer 2:

Reviewer #2: Some results and tables need to be update for correct cut point of RVNA

**Authors' comments:** *We really believe that following the cut-off points interpreted by Moore (2021)*, we are in agreement with the convention for individuals without previous vaccination, both for the contact indicator group and for the group considered to have adequate titers. The values have been corrected to ≥0.5 UL/ml instead >0.5 UL/ml as informed below.*

**Article referenced in our manuscript - reference no. 14.*

Reviewer #2: Conclusion need revision with better evidence

**Authors' comments:** *Thank you for your comment! We have added information throughout the manuscript in order to improve the evidence of the study. All changes are reported below and we hope that we have achieved the desired level of excellence.*

Reviewer #2: The use of the word "protective" refering to level of RVNA is incorrect, please delete all instances were that word was used, and use the correct expression: "adequate".
Also the adequate antibody level recommended by WHO is ≥0.5 UL/ml, in the manuscript is implied only >0.5 UL/ml, please correct, meaning table need to be redone also.

**Authors' comments:** *The reviewer is correct. Now all terms were corrected and we thank you for the valuable observation.*

**Now you read:** Throughout the manuscript, all the words “protective” have been replaced by “adequate” and all the values “>0.5/ above 0.5” have been replaced by “≥0.5/ equal to or above 0.5”.

Reviewer #2: The received manuscript is highly relevant and important for rabies science.
Unfortunately, the findings of rabies neutralizing antibodies (RVNA) in unvaccinated humans and dogs, even in healthcare workers are not properly supported by the data presented in the manuscript.

The claims in the article are very significant, and all efforts to demonstrate the accuracy of the statements and results need to be exhausted. The evidence about negative previous vaccination is lacking or insuficient, access to rabies vaccine, types of vaccines and years of use for humans and dog in the areas studied need to be explained in detail. If the individual with RVNA does not have a registry of his inmunization in a medical record, that does not rule out previous vaccination. Without a detailed explanation of vaccine delivery system, or the possibility for rabies vaccine access in other ways (such as: a campaign , outbreak reponse or due to work in a factory or industry that requires rabies immunisation and that is provided separately than the offical MoH services) we cannot conclude for sure the invididual was never vaccinated against rabies. A good discussion of those issues , biases and limitations is needed in the discussion section.
Another important issue is that , previous findings of rabies antibodies in local population (Gilbert et al 1992) did not find RVNA but rabies antibodies that were not neutralizing and levels of RVNA were not adecuate, therefore previous research does not support the findings in the current manuscript, then the presented findings would be really important and will dispute previous immuniological profiles published. To compare with previous results properly, the authors need to show  additional immunological test than only FVNA, of course additional differences on exposures and environmental factors in the population studied could lead to explain the findins, but that specific issues are not welll described in the manuscritp that even leads to canine exposures , and also is undefined regarding the type of bat exposures mentiones, and Brazil have serveral cycles for rabes in bats, and of course exposure factors are different, all that need to be well undesrtood in the
manuscript.   
Please discuss memory bias, and other potential issues that can lead to accept a dog or an indivudual as previously non vaccinated against rabies when was not. Same with healthcafre personnel.

I will be glad to review the changes and improvements for the manuscript, as I said, results are very significant given are well supported by evidence, and I sincerely hope the authors take the task and effort to provide what is needed to achieve publication.

**Authors' comments:** *We agree that the evidence on prior negative vaccination is non-existent or insufficient in the manuscript, so a paragraph has been added to clarify this point. Thank you for your comment.* *We know that there can be non-specific inhibition of viruses at low titers, even in unvaccinated individuals, however, it is worth noting that in humans and domestic animals the value of 0.5UI/mL is the cut-off adopted by both the WHO. In addition, it is worth mentioning the study by Moore (2021) already mentioned in this document as evidence of variability in the interpretation of cut-off points for individuals who have no previous prophylaxis. In any case, we are not aware of any articles by Gilbert et al. 1992 that might endorse this view. Also, when contacting Dr. Amy Gilbert, she confirmed that she does not have any papers published in 1992. She discussed the results of a publication by Lopez and others from 1992, where evidence of rabies antibodies in humans was previously documented. Specifically, she referenced the Lopez 1992 publication about antibody detection from 8 of 48 persons (one of the 8 later died) from two Amazonian communities affected by a rabies outbreak in 1990. Taken in context, the Lopez et al 1992 publication was an early and important report highlighting the routine nature of bites from vampire bats and the detection of naturally acquired serum rabies antibodies among humans in the Amazon region. However, it is important to point out that although they are within the same country, the Brazilian Amazon region differs completely from the central-western region of São Paulo state. Other points about the use of the correct cut-off point and the immunization of healthy individuals without previous vaccination were added throughout the manuscript in order to enhance and remedy the general notes.*

**Now you read:**

The seroneutralization techniques (RFFIT and FAVN) have been considered the main approved methods by the World Health Organization, which has adopted a very strict human cut-off, precisely covering possible inherent test interferences and considering the disease severity. In wild animals and other species, studies have considered less conservative cut-offs and indicated that values greater than 0.10IU/ml may indicate RABV virus circulation. However, the possibility of non-specific inactivation cannot be ruled out. (See lines 349-355).

The occurrence of rabies-specific antibodies in healthy, unvaccinated individuals may be the result of an alternative course of rabies virus exposure. Initially, Fekadu identified four alternative courses of rabies infection that could lead to detecting rabies antibodies in healthy individuals. (Please see lines 356-359).

When a host encounters a pathogen, antibodies that can specifically bind to the antigens on that pathogen are selected and increased in number. If the host survives such exposure, the enhanced immune response may be detected after pathogen elimination. Consequently, the presence of rabies-specific antibodies in healthy, unvaccinated individuals may indicate prior exposure to RABV with no fatal outcome. The presence of anti-rabies antibodies in unvaccinated individuals herein suggests prior viral exposure, but not necessarily active viral replication, as even a single dose of an inactivated rabies vaccine can induce anti-rabies responses. Although the natural development of rabies antibodies in healthy humans has been rarely documented, several studies have reported detectable rabies viral-neutralizing antibodies in serum from individuals living in highly endemic areas of rabies. (See lines 377-387).

The potential cross-reactivity among closely related viruses may also be a factor that makes interpreting rabies serology tests more complex, as their antigen similarity may enable antibody production against one virus to neutralize others. Since no other circulating lyssavirus has been reported in Brazil, rabies virus neutralizing antibodies may strongly suggest either a current infection or previous exposure to rabies virus. (See lines 402-407).

The Indigenous Health Secretariat has informed that the studied communities solely relied on the Secretariat for rabies prophylaxis protocol, with no independent pre- or post- exposure vaccination, confirming that vaccination cards reflected true vaccination status of all analyzed individuals. In addition, the São Paulo state suspended vaccination campaigns for dogs and cats since 2021. Finally, as people living in Indigenous communities may not fully understand the rabies risks, they are unlikely to seek medical assistance following suspicious bat contact, handling or bite. (Please see lines 415-422).
